# Limited English proficiency among adults with HIV in the United States – Medical Monitoring Project, 2015–2018

**DOI:** 10.1080/09540121.2020.1838428

**Published:** 2020-10-27

**Authors:** Mabel Padilla, Jennifer Fagan, Yunfeng Tie, John Weiser, Hanna B. Demeke, R. Luke Shouse

**Affiliations:** Division of HIV/AIDS Prevention, Centers for Disease Control and Prevention, Atlanta, GA, USA

**Keywords:** Limited English proficiency, HIV, HIV care, language services

## Abstract

Research suggests that language barriers in health care settings may adversely affect clinical outcomes and patient satisfaction. We describe the characteristics of adults with limited English proficiency (LEP) and diagnosed HIV in the United States. The Medical Monitoring Project is a complex sample survey of adults with diagnosed HIV in the United States that uses two-stage, probability-proportional-to-size sampling. We analyzed weighted interview and medical record data collected from June 2015–May 2018. The prevalence of LEP among adults with HIV was 10%. Higher percentages of adults with LEP, compared with adults with English proficiency (EP), were female, Hispanic/Latino, less educated and poor, only had Ryan White HIV/AIDS Program (RWHAP) health care coverage, attended RWHAP-funded facilities, were satisfied with their HIV medical care, were prescribed antiretroviral therapy (ART), were virally suppressed and received testing for sexually transmitted diseases. We found no statistical difference in ART adherence among adults with LEP and EP. Despite the association between LEP and the risk for health disparities, more persons with LEP were virally suppressed compared with persons with EP. One possible explanation is attendance at RWHAP-funded facilities by adults with LEP; however, future studies are needed to explore other possible explanations.

## Introduction

According to the 2015 American Community Survey, approximately 21% of people in the United States spoke a language other than English at home; of those, 41% had limited English proficiency (LEP) (U.S. Census Bureau). Persons with LEP accounted for 9% of the overall U.S. population in 2015 (Batalova & Zong, 2016). Research suggests that language barriers in health care settings adversely affect clinical outcomes and quality of care. Higher percentages of persons with LEP, compared with persons with English proficiency (EP), have reported difficulty understanding medication use, trouble communicating with providers, lower medication adherence, and lower patient satisfaction (Karliner et al., 2012; Morales et al., 1999; Wilson et al., 2005). These factors are negatively associated with retention in HIV care and adherence to antiretroviral therapy (ART), which are key determinants of HIV viral suppression (Dang et al., 2013; Paterson et al., 2000).

Previous studies have examined how LEP affects knowledge and receipt of HIV testing. One study found that adults with LEP were less knowledgeable about HIV testing recommendations, which may contribute to disparities in HIV testing and infection (Arya et al., 2013). In a study of Latinos with diagnosed AIDS, LEP was associated with late HIV testing (Wohl et al., 2009). Additionally, a comprehensive literature review found that LEP is a barrier to HIV testing by physicians (Burke et al., 2007). Despite the breadth of work on other patient populations with LEP, we found few studies specific to adults with LEP and HIV. To fill this gap, we present the only nationally representative estimates of the sociodemographic, behavioral, and clinical characteristics of adults with LEP and diagnosed HIV in the United States. We also describe differences between adults with LEP and EP.

## Materials and methods

The Medical Monitoring Project (MMP) is an annual cross-sectional survey designed to produce nationally representative estimates of the behavioral and clinical characteristics of adults with diagnosed HIV in the United States. Briefly, MMP used a two-stage sampling method. During the first stage, 23 jurisdictions were sampled from the United States, the District of Columbia, and Puerto Rico. During the second stage, simple random samples of persons with diagnosed HIV, aged ≥ 18 years, were drawn for each participating state or territory from the National HIV Surveillance System, a census of persons with diagnosed HIV in the United States. We analyzed pooled data from interviews and medical record abstractions collected during June 2015–May 2018.

Bilingual staff interviewed participants who primarily spoke Spanish using the Spanish-language version of the questionnaire. Interpreters were used for languages other than Spanish, or when a jurisdiction did not have bilingual staff. We excluded Puerto Rico from this analysis because English is not the main language spoken there; thus, our findings are limited to adults living in U.S. states (*N* = 11,371). MMP methods, including response rates, are described in detail elsewhere (Centers for Disease Control and Prevention [CDC], 2016). We weighted data to account for unequal selection probabilities and nonresponse.

### Measures

Respondents were asked “Do you speak a language other than English at home?” (“yes”, “no”) and “How well do you speak English?” (“very well, well, not well, not at all”). Respondents who spoke a language other than English at home and selected any option less than “very well” were classified as having LEP (*n* = 1,090). This question, a valid measure of English proficiency, has been the U.S. Census Bureau’s main survey question for assessing English proficiency (Vickstrom et al., 2015).

Sociodemographic variables included age, gender, race/ethnicity, education attainment, and whether respondents were born in the United States. Poverty level and health insurance coverage were reported for the 12 months before interview. Household poverty level was determined using the U.S. Department of Health and Human Services poverty guidelines for the calendar year about which the household income question was asked (U.S. Department of Health and Human Services [HHS], 2009).

We ascertained whether participants’ primary HIV care facility received any Ryan White HIV/AIDS Program (RWHAP) funding. Additionally, respondents were asked, “In general, how satisfied are you with the outpatient HIV medical care you received in the past 12 months?”. “Very satisfied” responses were classified as satisfied with HIV medical care and all other responses were classified as not satisfied. Respondents were asked about adherence to ART during the past 3 days using a validated three-item adherence scale (Wilson et al., 2014; Wilson et al., 2016). Respondents who reported not missing a dose during the past 3 days were classified as ART-adherent. Viral suppression, measured by the most recent viral load documented as undetectable or <200 copies/mL, was determined from medical records. Sexually transmitted disease (STD) testing was also determined from medical records; specifically, whether the respondent was tested for gonorrhea, chlamydia, and syphilis in the past year, as recommended in national guidelines (CDC, 2019). If testing for ≥1 of these STDs was not documented in the medical record, respondents were classified as not having received STD testing.

### Data analysis

We computed frequencies and weighted percentages describing characteristics of adults with diagnosed HIV and 95% confidence intervals (CIs) for these descriptive parameters. We used modified Rao-Scott chi-square tests to assess sociodemographic, behavioral, and clinical differences between adults with LEP and EP (*P* < .05 considered significant). We performed all analyses by using SAS 9.3 (SAS Institute, Cary, NC).

### Ethics statement

MMP data collection is part of routine public health surveillance and was determined to be non-research (CDC, 2010). Local institutional review board approval was obtained at participating states and territories when required. Informed consent was obtained from all interviewed participants.

## Results

Adults with LEP accounted for 9.6% (CI = 8.7–10.4) of all adults with diagnosed HIV in the United States (data not in table). Higher percentages of adults with LEP, compared with adults with EP, were 40–49 years old (33.7% vs. 23.8%), female (26.5% vs. 23.4%), Hispanic/Latino (79.4% vs. 13.1%), less than high-school educated (40.6% vs. 15.1%), living at or below the poverty level (54.6% vs. 41.1%), insured by RWHAP health care coverage only (23.2% vs. 7.3%) and born outside of the United States (82.1% vs. 7.4%). Higher percentages of adults with LEP, compared with adults with EP, received care at RWHAP-funded facilities (79.0% vs. 67.0%), were satisfied with their HIV care (83.7% vs. 80.3%), were prescribed ART (89.7% vs. 83.5%), were virally suppressed (77.5% vs. 69.6%), and received STD testing (49.9% vs. 33.4%). We found no statistically significant differences in ART adherence (Table 1).

## Discussion

Approximately one in ten adults with diagnosed HIV had LEP. Although adults with LEP lack resources to support health – including education, income, and health insurance – they were more likely than adults with EP to receive recommended HIV treatment and achieve viral suppression, which are both key to ending the HIV epidemic. A possible explanation for this seeming paradox is that a higher percentage of adults with LEP than adults with EP received care at RWHAP-funded facilities, which are twice as likely as other facilities to provide support services – including interpreter and social services – necessary for marginalized populations to achieve successful outcomes (Weiser et al., 2015). More than half of adults with LEP and HIV have incomes below the federal poverty level. People living in poverty are more likely to achieve viral suppression if they receive care at RWHAP-funded facilities (Weiser et al., 2015). The RWHAP – designed as a payer of last resort for high quality HIV care and treatment for low-income, uninsured and underinsured individuals and families – may mitigate some of the challenges to health faced by persons with LEP (U.S. Health Resources and Services Administration).

Title VI of the Civil Rights Act of 1964 protects persons with LEP from discrimination by requiring programs or institutions that receive Federal financial assistance – including RWHAP-funded facilities and other non-RWHAP-funded hospitals and clinics – to provide access to language services (HHS). Research shows that accessing a language-concordant physician or a professional interpreter substantially improves medication adherence, communication, and patient satisfaction (Karliner et al., 2007; Lee et al., 2002; Moreno & Morales, 2010). Thus, language might not be a barrier to seeking care and support for persons with LEP attending RWHAP-funded facilities.

Recent immigrants are more likely to have LEP (Wilson, 2014). The immigrant health paradox has often been used to explain better health outcomes among immigrants compared with persons born—or having spent more time—in the United States (Teruya & Bazargan-Hejazi, 2013). Future studies may explore the role that language and other measures of acculturation—e.g., Length of time in the United States, acculturative stress, immigration status—play in better HIV clinical outcomes.

These results were subject to several limitations. The behavioral data were self-reported, thus subject to social desirability and recall bias. Our data are cross-sectional; thus, causality cannot be inferred. Our analysis only includes adults aware of their HIV diagnosis; therefore, our estimates of LEP may be lower than what might be found among all persons with LEP and HIV. As noted earlier, LEP is a barrier to HIV testing and has been associated with late HIV testing; therefore, more adults with LEP may have undiagnosed HIV compared with adults with EP and thus may be excluded from MMP (Burke et al., 2007; Wohl et al., 2009). Furthermore, we did not measure access to language services among persons with LEP.

Despite the association between LEP and the risk for health disparities, the clinical outcomes for adults with LEP were better than those of persons with EP—but still suboptimal. One possible explanation is that a higher percentage of adults with LEP attended RWHAP-funded facilities. Access to health care with substantial support services is critical to ensuring positive health outcomes. Future studies might explore the role that facility type and acculturation plays in better viral suppression among adults with LEP compared with adults with EP.

## Figures and Tables

**Table 1.** Limited English proficiency among adults with diagnosed HIV – Medical Monitoring Project, 2015–2018.

	Total (*n* = 11,371)		Limited English Proficiency (*n* = 1,090)		English Proficiency (*n* = 10,281)		Rao-Scott chi-square *P* value
Characteristics	Weighted %	CI	Weighted %	CI	Weighted %	CI	
*Age in years*							*P* < 0.001
18–29	9.1	8.3–9.9	4.3	2.8–5.9	9.6	8.8–10.5	
30–39	16.8	16.0–17.7	20.1	17.3–22.9	16.5	15.6–17.4	
40–49	24.7	23.7–25.8	33.7	30.6–36.7	23.8	22.7–24.9	
≥50	49.3	47.9–50.7	41.9	38.4–45.5	50.1	48.6–51.6	
*Gender*							*P* = 0.032
Male	74.8	73.2–76.4	71.1	67.7–74.4	75.2	73.4–77.0	
Female	23.7	22.0–25.3	26.5	23.1–29.8	23.4	21.6–25.2	
Transgender	1.5	1.2–1.8	2.5	1.2–3.7	1.4	1.1–1.7	
*Race/ethnicity* ^a^							*P* <0.001
White, non-Hispanic	30.8	27.2–34.3	2.5	1.6–3.4	33.8	29.7–37.8	
Black, non-Hispanic	42.6	37.8–47.4	13.2	10.7–15.8	45.7	40.6–50.8	
Hispanic/Latino	19.5	18.0–20.9	79.4	76.1–82.7	13.1	11.8–14.4	
Other	7.2	6.3–8.1	4.9	3.1–6.7	7.5	6.5–8.4	
*Education*							*P* <0.001
< High school	17.6	16.3–18.9	40.6	37.2–44.1	15.1	13.7–16.6	
High school diploma or equivalent	25.6	24.5–26.8	25.0	22.2–27.8	25.7	24.5–27.0	
>High school	56.8	55.0–58.6	34.4	31.2–37.6	59.1	57.1–61.2	
*Poverty*							*P* <0.001
Above federal poverty level	57.6	55.4–59.8	45.4	41.5–49.3	58.9	56.5–61.3	
At or below federal poverty level	42.4	40.2–44.6	54.6	50.7–58.5	41.1	38.7–43.5	
*Health insurance coverage during the last 12 months*							*P* <0.001
Yes	89.3	87.5–91.1	74.8	70.7–78.8	90.8	89.0–92.7	
Uninsured	1.9	1.4–2.5	2.0	0.5–3.5	1.9	1.3–2.5	
Uninsured (RWHAP only)	8.8	7.2–10.3	23.2	19.1–27.4	7.3	5.7–8.8	
*Born in the United States*							*P* <0.001
Yes	85.5	84.4–86.7	17.9	15.1–20.8	92.6	91.8–93.5	
No	14.5	13.3–15.6	82.1	79.2–84.9	7.4	6.5–8.2	
*Attended RWHAP-funded facility*							*P* <0.001
Yes	68.2	62.7–73.7	79.0	74.5–83.6	67.0	61.2–72.9	
No	31.8	26.3–37.3	21.0	16.4–25.5	33.0	27.1–38.8	
*Satisfied with outpatient HIV medical care*							*P* = 0.019
Yes	80.6	79.7–81.6	83.7	81.2–86.2	80.3	79.3–81.3	
No	19.4	18.4–20.3	16.3	13.8–18.8	19.7	18.7–20.7	
*ART adherent (100% dose adherence in previous 3 days)* ^b^							*P* = 0.986
Not adherent	56.3	55.1–57.6	56.4	52.8–60.0	56.3	55.0–57.7	
Adherent	43.7	42.4–44.9	43.6	40.0–47.2	43.7	42.3–45.0	
*Prescribed ART*							*P* = 0.001
Yes	84.1	82.6–85.6	89.7	87.1–92.3	83.5	81.9–85.1	
No	15.9	14.4–17.4	10.3	7.7–12.9	16.5	14.9–18.1	
*Viral suppression*							*P*=<0.001
Most recent viral load undetectable or	70.4	68.4–72.3	77.5	74.1–80.9	69.6	67.7–71.6	
<200 copies/mL							
Most recent viral load documented detectable, ≥200 copies/mL or missing/unknown	29.6	27.7–31.6	22.5	19.1–25.9	30.4	28.4–32.3	
*Received STD testing*							*P* = <0.001
Yes	35.0	33.5–36.5	49.9	46.3–53.4	33.4	31.8–34.9	
No	65.0	63.5–66.5	50.1	46.6–53.7	66.6	65.1–68.2	

All percentages are weighted. *P* values were calculated by using the Rao-Scott chi-square test.

All percentages are weighted. *P* values were calculated by using the Rao-Scott chi-square test.

a Race and ethnicity are mutually exclusive. Hispanic/Latinos could be of any race.

b Denominator is persons currently taking ART (*n* = 11,209).
